# Combined effects of temperature and the herbicide diuron on Photosystem II activity of the tropical seagrass *Halophila ovalis*

**DOI:** 10.1038/srep45404

**Published:** 2017-03-30

**Authors:** Adam D. Wilkinson, Catherine J. Collier, Florita Flores, Lucas Langlois, Peter J. Ralph, Andrew P. Negri

**Affiliations:** 1 College of Marine and Environmental Sciences, James Cook University, Townsville, Queensland, Australia; 2 Australian Institute of Marine Science, Townsville, Queensland, Australia; 3 Centre for Tropical Water & Aquatic Ecosystem Research (TropWATER), James Cook University, Cairns, Queensland, Australia; 4 Climate Change Cluster (C3), University of Technology Sydney, New South Wales, Australia

## Abstract

Tropical seagrasses are at their highest risk of exposure to photosystem II (PSII) herbicides when elevated rainfall and runoff from farms transports these toxicants into coastal habitats during summer, coinciding with periods of elevated temperature. PSII herbicides, such as diuron, can increase the sensitivity of corals to thermal stress, but little is known of the potential for herbicides to impact the thermal optima of tropical seagrass. Here we employed a well-plate approach to experimentally assess the effects of diuron on the photosynthetic performance of *Halophila ovalis* leaves across a 25 °C temperature range (36 combinations of these stressors across 15–40 °C). The thermal optimum for photosynthetic efficiency (▵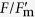) in *H. ovalis* was 31 °C while lower and higher temperatures reduced ▵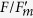 as did all elevated concentrations of diuron. There were significant interactions between the effects of temperature and diuron, with a majority of the combined stresses causing sub-additive (antagonistic) effects. However, both stressors caused negative responses and the sum of the responses was greater than that caused by temperature or diuron alone. These results indicate that improving water quality (reducing herbicide in runoff) is likely to maximise seagrass health during extreme temperature events that will become more common as the climate changes.

Coastal seagrass meadows are ecologically important habitats globally and occur along all coasts of Australia^1^. As primary producers, seagrasses form the basis of food webs and almost exclusively comprise the diet of important macro-grazers, such as dugongs (*Dugong dugon*) and green sea turtles (*Chelonia mydas*)^2^. Additionally, seagrass meadows act as nursery and foraging grounds for many subsistence and commercial fishery species^3^,^4^,^5^. The extensive network of roots and rhizomes below ground also facilitate sediment stabilisation^1^,^6^ and sequester carbon, which is highly valued in carbon trading (known as “Blue carbon”)^7^,^8^. Seagrass leaves incorporate inorganic nutrients and filter suspended organic matter from the water column, improving coastal water quality^9^,^10^. In the tropics, seagrasses also aid the primary production of adjacent coral reef ecosystems through energetic and material subsidies^11^.

Water temperature drives seasonal variations in growth rates of seagrasses^12^,^13^ and contributes to species-specific geographical distribution^14^,^15^. In shallow habitats such as inshore seagrass meadows, water temperatures can considerably exceed daily or seasonal averages during low tide events (for a few hours), whereby ponded waters are heated beyond that of the ocean^16^,^17^,^18^,^19^. These elevated temperatures cause thermal stress, particularly to the photosynthetic apparatus^14^,^19^,^20^,^21^,^22^,^23^. Photosynthetic efficiency increases with temperatures approaching the thermal optimum for photosystem II (PSII) activity, but they rapidly decline at temperatures exceeding the optimum^21^,^22^,^24^. PSII in particular, has been identified as one of the most heat-sensitive photosynthetic units in higher plants^25^,^26^,^27^. Heat-induced decline in photosynthesis is also related to disruption of the stability of chloroplasts and the detachment of the light harvesting complex from the PSII reaction centre of terrestrial plants^20^,^24^,^28^,^29^. The optimum temperature for efficiency of PSII is between 35 and 40 °C in a number of tropical-distributed seagrass species^19^,^30^. However, more broadly distributed (temperate-tropical) species, such as *Halophila ovalis*, appear to be more sensitive to thermal stress than other tropical species^19^,^21^,^30^. Some tropical seagrasses are already living near the edge of their thermal limits with optimal temperatures being frequently surpassed during day time low tides^19^, and the frequency and severity of these extreme events will rise due to increases in both air and sea temperature^31^.

The summer monsoon season in the tropics results in frequent flood plume events and increased coastal turbidity^32^ which can either exacerbate or ameliorate the impact of thermal stress on seagrasses in the inshore Great Barrier Reef (GBR) lagoon^14^. In addition to light limitation, flood plumes in the GBR are responsible for transporting agricultural pesticides from the catchments into the lagoon^33^,^34^,^35^,^36^. The most common pesticides detected on the GBR are the PSII herbicides, including diuron and atrazine, which occur at some inshore sites year-round^37^, but usually peak in concentration during the summer monsoon season^33^. PSII herbicides bind reversibly to the D1 protein of the thylakoid membrane and therefore directly supress linear electron transport in PSII^38^,^39^. PSII herbicides are designed to inhibit function and damage PSII in terrestrial weeds, however since the D1 protein in PSII is conserved among plants, non-target species, such as seagrasses and corals, are equally affected^40^,^41^,^42^,^43^. Plant death occurs from chronic starvation (long-term electron transport rate inhibition) under moderate irradiance^41^ or oxidative stress under higher irradiance^44^.

Chlorophyll fluorescence techniques have often been used to measure the effects of herbicides and thermal stress on electron transport in PSII in seagrasses^40^,^41^,^42^,^43^,^45^. In illuminated plants, photochemical quenching (transfer of electron energy, ultimately leading to ATP production) takes place when PSII reaction centres are functioning optimally. To protect against photo-oxidative damage, photo-protective processes dissipate excess energy as either heat or chlorophyll *a* fluorescence^44^. Chlorophyll *a* fluorescence can be measured using Pulse Amplitude Modulated (PAM) fluorometry, a non-invasive technique applied to measure photochemical efficiency^46^,^47^ and impacts on electron transport and damage to PSII can therefore be inferred. The two most common and sensitive end points which are tested regularly in photophysiology studies are effective quantum yield (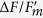) and maximum quantum yield (*F*_*v*_/*F*_*m*_)^47^,^48^. In the presence of PSII herbicides, 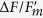 inhibition indicates reduced photosynthetic efficiency^47^,^48^ and is the most sensitive assessment of PSII herbicide impacts on photosynthesis at a given light intensity^47^. Long term inhibition of 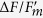 by PSII herbicides (such as diuron) exposure can lead to a decline in stored energy and seagrass mortality^41^. Other studies have also demonstrated PSII herbicide exposure reduces seagrass survival^49^,^50^ and growth^49^,^51^. Reduced *F*_*v*_/*F*_*m*_ caused by herbicide exposure or thermal stress is proportional to PSII damage, and indicates the likelihood of a prolonged recovery of PSII efficiency after the stress has been removed^47^,^48^.

Herbicide concentrations peak during the summer monsoon season within the nearshore GBR^36^,^52^ and this coincides with peak summer ocean temperatures as well as temperature extremes in shallow seagrass meadows^53^. Thus, thermal stress can occur simultaneously with peak herbicide exposure during the summer monsoon. Since both stressors act on the processes and/or integrity of PSII (see above), their combined effects can be assessed using chlorophyll fluorescence techniques. Recent studies which applied PAM fluorometry have demonstrated that the presence of PSII herbicides increases the vulnerability of tropical symbiotic organisms such as corals^54^ and foraminifera^55^ to thermal stress. However, tropical seagrasses are more likely to be exposed to herbicides in coastal runoff than coral reefs^33^, and so far there have been no studies examining the combined effects or potential interactions of thermal stress and herbicides on seagrass physiology and health.

Here we test the hypothesis that simultaneous acute exposures of the broadly-distributed seagrass species *H. ovalis* to thermal stress and diuron will affect PSII to a greater extent than either stressor in isolation. A miniature bioassay^45^ was applied to measure acute responses of PSII function of tropical seagrass, *H. ovalis* to 36 combinations of diuron (concentrations 0–30 μg l^−1^) and temperature (15–40 °C). The effect of thermal stress coupled with diuron exposure on photosynthetic performance (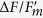) of isolated leaves was assessed in concentration-response experiments over a 24 h exposure period in a static system. Recovery of PSII activity (*F*_*v*_/*F*_*m*_) was assessed after a further 24 h at 30 °C (control temperature) in uncontaminated seawater. Understanding how these effects combine additively, antagonistically or synergistically will improve our understanding of whether local management of pollution (such as herbicides) can reduce the vulnerability of seagrasses to annual thermal stress events and global change.

## Results

### Effects of temperature alone on photosystem II processes

**Figure 1:**
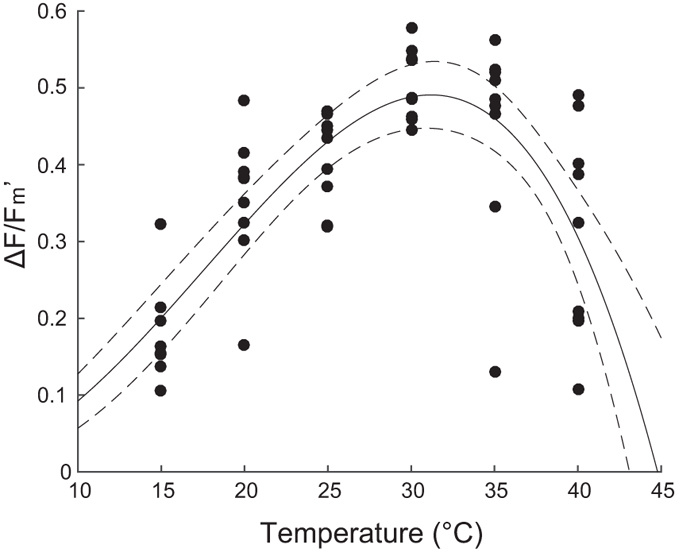
Effect of 24 h exposure to a range of temperature on 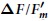 of *H. ovalis* in the absence of diuron. All 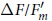 measurements were conducted under 100 μmol photons m^−2^s^−1^ irradiance. Mean ± SE of nine replicate leaf samples. Dashed lines represent ±95% CI. A three parameter model was fitted to the 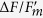 data according to Adams *et al*.^84^.

Temperature alone had a strong effect on effective quantum yield (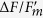) of *H. ovalis* leaves after 24 h exposures in the absence of diuron (Fig. 1). 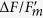 reached a modelled optimum of 0.49 ± 0.6 (95% CI) at 31.2 °C ± 0.6 °C. The maximum measured 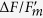 of 0.50 ± 0.02 (SE) was recorded at 30 °C, dropping to 0.18 ± 0.02 at 15 °C and 0.31 ± 0.05 at 40 °C in the absence of diuron.

### Effects of simultaneous exposure of seagrass to diuron and thermal stress

**Figure 2:**
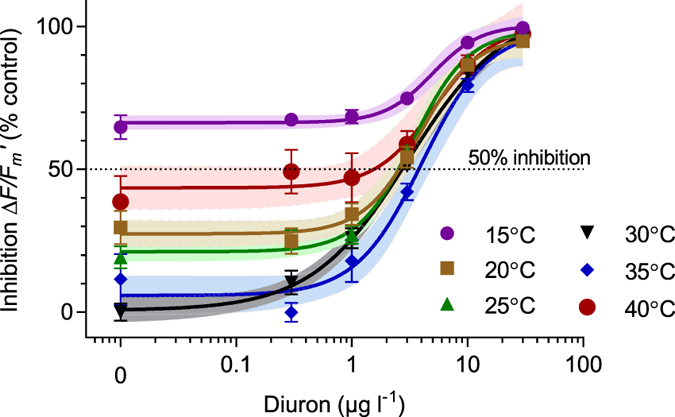
Concentration-response curves (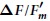) for diuron with *H. ovalis* at six temperatures. 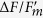
 inhibition relative to 30 °C solvent control. All 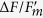 measurements were conducted under 100 μmol photons m^−2^s^−1^ irradiance. Mean ± SE of nine replicate leaf samples. Shaded areas represent the 95% confidence boundaries of each 4 parameter sigmoidal model.

The 30 °C treatment was designated as the “control” temperature and inhibition of 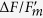 was calculated relative to fresh seawater at 30 °C. With increasing diuron concentrations, greater inhibition of 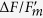 was observed (Fig. 2). A 50% inhibition of 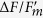 (IC_50_) at 30 °C was 2.9 μg l^−1^ diuron (Table 1). The strong effect of temperature alone on 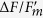 was apparent in the solvent control and 0.3 μg l^−1^ treatments with inhibition of effective quantum yield by over 40% at the extreme temperatures of 15 °C and 40 °C. As diuron concentrations increased, the inhibition of 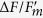 in each of the temperature treatments converged and the IC_50_s of seagrass in the 20 °C–30 °C were similar. No IC_50_ was reported for the 15 °C treatment as inhibition was greater than 50%, even in the absence of diuron. While the sensitivity of PSII to diuron was lowest at 35 °C (highest IC_50_ of 4.1 μg l^−1^), a 5 °C increase in temperature yielded a much lower IC_50_ of 1.4 μg l^−1^ (Table 1).

**Figure 3:**
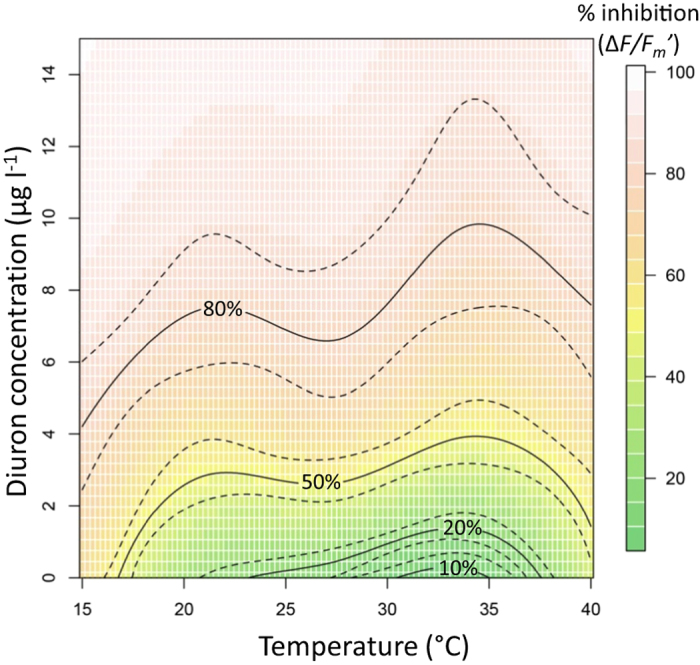
Modelled relationship between temperature, diuron concentration and inhibition of 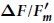. The R code for this quasibinomial generalized additive model (GAM) best fit model can be found in Table S1. Dashed lines are 95% confidence intervals based on the standard errors calculated by the predict.gam function (see “mgcv” package R documentation for details).

Exposing the seagrass to 36 combinations of temperature and diuron enabled us to explore the relationship between these stressors and resulting effects on photosynthetic performance. The effect of temperature and diuron concentration on the inhibition of 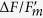 was modelled using a generalized additive model in R (Fig. 3). The quasibinomial model with a logit link function exhibited an r^2^ of 0.87, explaining 84% of deviance. The data code for R and the % inhibition data are in Table S1, and these allows the calculation of inhibition of 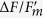 for any given combination of temperature and diuron. The model was used to predict a continuum of IC_50_ values which were greatest over the optimal temperature range (30 °C–35 °C) for photosynthetic performance, confirming the greater sensitivity of photosystems to diuron at extreme low and high temperatures.

### Interactive effects of simultaneous temperature and diuron exposure on photosystem II

**Figure 4:**
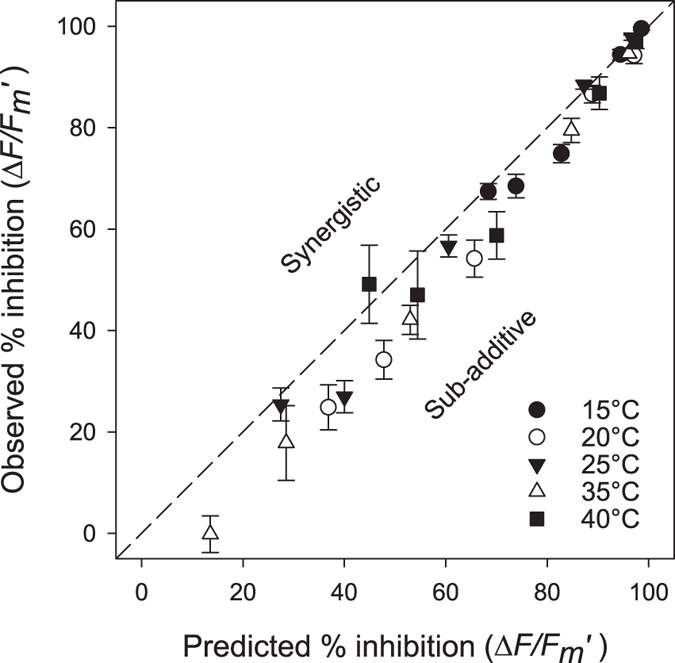
Comparison between predicted (Response Addition) and observed inhibition of 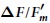 in response to combinations of temperature and diuron. Data points intersecting the zero-interaction line (1:1) indicate additivity; points below the additivity line suggest sub-additivity; and data points above the additivity line indicate synergism. Inhibition calculated relative to 30 °C solvent control mean. Mean ± SE of nine replicate leaves.

The two-way ANOVA confirmed the significant effects of temperature and diuron on 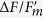 (Table 2). The interaction between these two stressors was also highly significant (p < 0.01) indicating that the effects of temperature and diuron were not additive. To investigate the type of interaction we plotted the 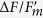 inhibition expected for additivity (according to the response addition (RA) model which assumes independent modes of action) versus observed inhibition (Fig. 4). Many of the measured inhibition data points fell below the 1:1 line expected for additive impacts (Fig. 4). This figure demonstrates that the interaction between temperature and diuron was sub-additive or antagonistic for most of the combinations of stressors, especially at low inhibition levels. At high inhibition levels (above 80%) most of the data lies on the 1:1 line, indicating additivity.

### Recovery of function of PSII in leaves after the exposures

**Figure 5:**
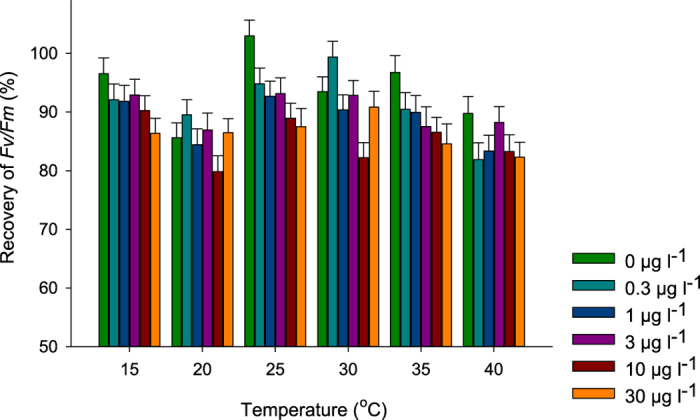
Recovery in *Fv*/*Fm* of *H. ovalis* in fresh seawater at 30 °C 24 h after the thermal and herbicide stress exposures. Data is plotted as the mean % recovery of individual leaves in comparison to measurements before the start of the exposures.

Recovery of PSII function (*Fv*/*Fm)* in the leaves following exposure to 36 combinations of temperature and diuron was significantly affected by both temperature and exposure to diuron (Fig. 5, Table 3). Recovery was lowest for leaves exposed at 40 °C and/or higher diuron concentrations; however recovery was generally good (>80% in comparison to the start of the exposures) and there were no significant interactive effects on recovery between these stressors (Table 3).

## Discussion

This study represents the first investigation into the simultaneous acute effects of temperature and herbicide on the photosystem of seagrass. The miniature well plate approach enabled the effects of 36 combinations of these stressors to be assessed across a 25 °C temperature range (15–40 °C). The thermal optimum for photosynthetic efficiency (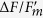) in *H. ovalis* was ~31 °C (fitted) while low and high temperatures inhibited 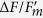 as did all elevated concentrations of diuron. There were significant interactions between the effects of temperature and diuron, with a majority of the combinations of these stressors causing sub-additive (antagonistic) effects. While the combined stress was generally less than additive, photosystems were more sensitive to diuron at temperatures outside the expected growth range of 20–35 °C.

### Effects of temperature alone on photosystem II processes

The geographic distributions of seagrasses are influenced by species-specific thermal optima, with long-term survival of tropical seagrass species generally occurring at a thermal range from 15 °C to 33 °C^19^,^56^,^57^. Here the thermal optimum (modelled) for photosynthetic performance of PSII in *H. ovalis* (maximum 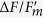 was 31 °C, Fig. 1) was slightly higher than average summer temperature within the meadow from which it was collected (29.4 °C in December to February from 2005 to 2015)^53^. However, the maximum daily temperature exceeded 31 °C in summer (December to February, from 2005–2015) for at least 30 minutes on 52% of days^53^. 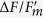 declined considerably at temperatures above 35 °C, which has been exceeded on 8% of summer days (from 2005 to 2015) suggesting reduced photosynthetic activity due to thermal stress occurs in summer at the collection site. Reduced activity in PSII (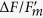 and *F*_*v*_/*F*_*m*_) as well as photochemical and non-photochemical quenching outside the normal thermal range in temperate *H. ovalis* were also reported over 4 d exposures^21^, and in repeated 4 h exposures to ≥35 °C in three tropical Australian species, including *H. ovalis*^24^. Campbell, *et al*.^24^ also demonstrated that four other species, which have distributions more restricted to warmer sub-tropical to tropical waters, were more tolerant to thermal stress. *H. ovalis*, with a distribution from temperate to tropical regions is likely to live close to its upper thermal tolerance in the tropics and intertidal plants may therefore be more vulnerable to spikes during summer low-tide events when water temperatures can increase by 10 °C over short periods to 40 °C^19^,^58^.

The sensitivity of photosynthesis to high temperature stress is likely due to the heat liable nature of the PSII apparatus^56^,^59^,^60^. Impacts can include protein denaturing^15^,^61^,^62^, alteration of thylakoid membrane conformation^20^,^21^ and disassociation of the light harvesting complex from PSII^29^. Changes in the performance of PSII is therefore likely to represent an effective early indicator of thermal stress compared with many other indicators^18^. Impacts on photosynthetic processes in turn disrupt carbon balance, where increased respiration, relative to photosynthetic output results in reduced carbon production^21^,^57^,^63^,^64^,^65^. To assess whether the PSII in *H. ovalis* remained intact after the exposure, *F*_*v*_/*F*_*m*_ measurements (an indicator maximum PSII activity) were taken in fresh seawater at 30 °C prior to the 24 h temperature and diuron exposures and again following recovery in fresh seawater for a further 24 h. Recovery of *F*_*v*_/*F*_*m*_ in *H. ovalis* following temperature exposure (in the absence of diuron) was generally consistent across the 25 °C temperature range, but was lower for leaves exposed to 40 °C (Fig. 5). This indicates that temperatures above 35 °C may represent an important upper thermal threshold as reduced photosynthetic capacity and some limitation in recovery suggests moderate irreversible damage to photosystems^21^,^24^. Furthermore, these extreme elevated temperatures are reached rapidly (within hours) in tropical seagrass meadows^19^,^53^,^66^, which may not provide time for acclimation. In all other treatments strong recovery of *F*_*v*_/*F*_*m*_ indicate little irreversible damage. Less is known about chilling effects on PSII in seagrass; however Ralph^21^ reported similar results with temperate *H. ovalis* exposed to low temperatures of 10 °C and 12.5 °C. In that study, steady F_o_ in *H. ovalis* under chilling conditions may have been associated with thermal deactivation of PSII reaction centres. Importantly, the thermal optima and the lower and upper thresholds for thermal stress on the photosystems of *H. ovalis* (or other seagrass species) are likely to differ due to acclimation to the local climate. Studies where the seagrass is more slowly acclimated to these lower temperatures are required to determine whether this effect on photophysiology is likely to be encountered in the field.

### Effects of diuron alone on photosystem II processes

Diuron inhibited 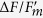 in *H. ovalis* by 50% at 2.6 μg l^−1^ at the control temperature of 30 °C (Fig. 2A, Table 1). This result was within the same range as previous studies examining effects on isolated leaves of *H. ovalis* (3.5–4.3 μg l^−1^)^43^,^45^ and potted *H. ovalis* (3.0 μg l^−1^)^45^, *H. uninervis* (2.4–2.8 μg l^−1^) and *Z. muelleri* (2.4–2.5 μg l^−1^)^40^,^41^. Inhibition of 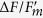 is one of the most sensitive indicators of stress by PSII herbicides on seagrasses and other plants as the change in fluorescence signal (increase in *F*_*o*_) represents a direct response of the closure of the electron transport mechanism by the binding of herbicide molecules to the D1 protein^47^,^67^. The 24 h exposures to diuron here: (i) ensured maximum uptake through the leaves and impact on PSII^45^; (ii) allowed enough time to measure impacts of thermal stress (Fig. 1); and (iii) was short enough to also allow for an additional set of recovery measurements to be made at 48 h without PSII deterioration of these detached leaves (Fig. 5). While the effects of this short exposure to diuron caused the maximum impact on 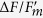
45, the toxicity of diuron (IC_50_) in seagrass has been shown to remain consistent over much longer exposures of 11 weeks^41^. Reduced photosynthetic capacity in seagrass from long-term exposures to PSII herbicides has flow-on impacts which include reduced carbon storage in the root-rhizome, reduced growth, and survival^41^. Tropical, nearshore habitats of the GBR that include seagrass meadows are likely to be periodically exposed to PSII herbicides at concentrations greater than current guidelines^68^. While seagrasses are not expected to be exposed to the concentrations of diuron that had a 50% impact on 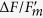, a wide range of diuron concentrations were applied to enable accurate quantification of the lowest toxic thresholds (IC_10_ values) which are important for guideline development. The toxic threshold IC_10_ = 0.4 μg l^−1^ represents an environmentally relevant scenario, with diuron concentrations above 0.9 μg l^−1^ discharging into nearshore areas of the GBR lagoon, particularly in the Mackay Whitsunday region^33^. The influence of co-stressors such as temperatures outside the normal thermal range of *H. ovails* (see above) influences the sensitivity of seagrass to diuron (see below).

### Interactive effects of simultaneous temperature and diuron exposure on photosystem II

The combined effects of temperature and diuron in 36 combinations over 24 h on the performance of PSII in *H. ovalis* was assessed in a variety of ways. The effects of temperature on the ecotoxicological diuron concentration-response curves were clear (Fig. 2), with each curve outside the range 30°–35 °C being shifted upwards (greater inhibition of 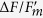) indicating a strong negative effect of temperature extremes on 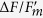. The exposure to thermal stress also affected the sensitivity to diuron with leaves exposed to the more extreme temperatures exhibiting lower IC_50_s 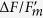 (Table 1). The concentration-response curves were not parallel, and this convergence at higher diuron concentrations indicates interactions between temperature and diuron on 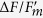
69. This was confirmed by the 2-way ANOVA revealing strong interactions (p < 0.01) between these stressors (Table 2). A comparison of the observed against the predicted effects of combinations of temperature and diuron according to the Response Addition model revealed most of the combinations caused less inhibition of 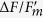 than would be expected if the responses were additive (Fig. 4). This represents an antagonistic interaction across most of the stressor combinations^69^, but since stressful temperatures and diuron exposure both caused negative responses and the sum of the responses was generally greater than that caused by temperature or diuron alone (Fig. 4), we consider “sub-additivity” to be a more appropriate description for this interaction (some other forms of antagonism can reverse or cancel individual effects)^70^. The GAM (Fig. 3) which described the relationship between temperature and herbicide impacts on 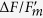 also demonstrated how combinations of temperature extremes and diuron exposure cause the greatest inactivation of PSII. The GAM enables inhibition to be predicted for any combination of temperature and diuron (within the tested range) to be calculated. This has application in plant growth models that build predictions based on multiple environmental conditions, and for the interpretation of routine seagrass health monitoring where both temperature and diuron exposure are known. Previously the effects of diuron and thermal stress has been shown to cause additive effects on 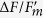 in symbiotic corals^41^ and their isolated symbionts^71^ and in foraminifera^55^. Although outcomes for PSII activity in seagrass was also worse for combinations of temperature stress and herbicide exposure, the sub-additivity reported here may reflect a greater flexibility in PSII function across a wider thermal range as seagrasses are generally considered far more tolerant of thermal stress than corals and foraminifera^72^. The recovery of PSII (as indicated by *F*_*v*_/*F*_*m*_, Fig. 5) in leaves exposed to higher herbicide concentrations near the thermal maximum was not as great as those exposed to lower diuron concentrations, indicating potential chronic damage to PSII at diuron concentrations above 3 μg l^−1^. However, in general, recovery was good and temperature had a greater influence on the recovery of function in PSII.

Temperature affects thylakoid membrane characteristics (including binding site configuration and conformation) and rates of diffusion of toxicants^73^,^74^. As a result, sub-optimal temperatures (both low and high) may cause reduced binding efficiency of diuron and thus change sensitivity to PSII herbicides, explaining the reduced IC_50_s at 20 °C and 40 °C. From another perspective, the presence of diuron (or other PSII herbicides) is likely to narrow the optimal temperature range for seagrass, resulting in stress responses at lower maximum temperatures, as reported for corals and foraminifera^54^,^55^,^71^. Throughout the GBR lagoon and catchment area seasonally high sea surface temperatures coincide with the monsoonal flood plume events and the highest herbicide concentrations are detected near seagrass habitats under these conditions^33^. The potential for inshore and estuarine seagrasses to simultaneously experience thermal stress and herbicides (as well as irradiance and low salinity osmotic stress) are high during this period and cumulative impacts on seagrass populations are likely. Similarly, temperate seagrass species (which have lower thermal optima), are often exposed to extreme variations in temperature between seasons^15^ and can also be exposed to contaminants including PSII herbicides^75^. While these scenarios are recognised by management agencies and regulators^76^, more studies are needed to quantify these complex interactions^77^ to effectively assess the risks posed by cumulative stressors to seagrass meadows and to inform future water quality guidelines to protect tropical seagrass species in a changing climate.

## Methods

### Herbicides

Diuron (3-(3,4-dichlorophenyl)-1,1-dimethylurea) is one of the most commonly detected PSII herbicides within the lagoon and nearshore waters of the GBR^33^,^36^,^78^, and is a persistent contaminant in seawater with a half-life of over 130 days^79^,^80^. Working solutions of diuron (Sigma Aldrich, >95% pure) were prepared in filtered seawater (0.45 μm) using ethanol as the solvent carrier (<0.03% v/v). A series of five diuron concentrations (0.3, 1, 3, 10, 30 μg l^−1^) were tested as well as a solvent control treatment (0 μg l^−1^). Nominal concentrations are reported in this study as diuron is non-volatile and has a water solubility >40 mg l^−1^ and octanol-water coefficient (log K_ow_) <3 making loss to adsorption on test vessels unlikely^81^. The ranges of salinity (32–34 psu), pH (8.1–8.2) and dissolved oxygen (>7.5 mg l^−1^) did not change throughout the exposures.

### Sample collection

*Halophila ovalis* is a colonising seagrass species in tropical and temperate marine habitats throughout Australia^82^. It grows rapidly with leaf pairs emerging from the rhizome and is considered sensitive to environmental stress^72^. *H. ovalis* plants were collected haphazardly across intertidal meadows during low tide from Cockle Bay, Magnetic Island (19° 10.88′S, 146° 50.63′E) under permit MTB41, a permit issued for limited impact research in the GBR Marine Park which was assessed through the Department of Employment, Economic Development and Innovation self-assessable Fisheries Queensland Code MP05 for the removal of marine plants. The seagrass used in the study was collected in June 2015 and was not exposed to appreciable concentrations of PSII herbicides. The maximum concentration of PSII herbicides over the preceding year at Magnetic Is was <0.01 μg l^−1 ^83, well below concentrations that affect Photosystem II^41^. A small core (10 cm) of seagrass with its associated sediment (5–10 cm depth) was removed and placed in plastic plant pots lined with plastic bags. Seawater was added to the bag and secured to minimise loss of humidity and for transport purposes. Plants were transported to the Australian Institute of Marine Science (AIMS), Townsville, Queensland, and placed into 1000 l aquaria within 4 h from collection under moderate light intensity (270–300 μmol photons m^−2^s^−1^) and ambient water temperature conditions (28 ± 1 °C).

### Leaf preparation and screening

To ensure only healthy leaves were used in the well plate experiments, isolated leaves underwent a screening process^45^. Before removing leaves, any epiphyte growth was removed from the leaf surface. Second and third leaf pairs from the terminal, apical end of the rhizome were selected and removed with scissors. Stems were cut to the base of the leaf and pinched closed with forceps to minimise formation of air bubbles in the midrib. Single leaves were placed into individual wells of 12-well plates (Nunclon, Thermo scientific) containing 0.45 μm-filtered seawater (5 ml in each well). Leaves were dark-adapted for 30 min and the maximum quantum yield (*F*_*v*_/*F*_*m*_) of each leaf was measured using the Imaging-PAM (see below for details) and only leaves exhibiting *F*_*v*_/*F*_*m*_ greater than 0.65 were selected for the assays^45^. Average leaf length was 10.0 mm ± 2.5 (range of all leaves) and width was 4.8 mm ± 1.2.

### Chlorophyll a fluorescence

Chlorophyll *a* fluorescence was measured using an Imaging-PAM (I-PAM, Walz GmbH, Germany). The treatment temperatures were maintained during measurement process by placing well plates (one at a time) in a custom heating block/water bath system under the I-PAM imaging chamber. Data-MAXI software (Imaging Win, Walz GmbH, Germany) was used to select a single area of interest (AOI) of 3–5 mm diameter for each leaf for fluorescence measurements. Minimum fluorescence (*F* with illuminated samples and *F*_*0*_ with dark-adapted samples) was initiated and recorded by applying a weak pulse-modulated red measuring light (650 nm, 0.15 μmol photons m^−2^s^−1^). To quantify light-adapted maximum fluorescence (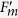) a short pulse (800 ms) of saturating actinic light (>3000 μmol photons m^−2^s^−1^) was applied and the effective quantum yield of PSII calculated from 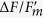 = 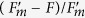. Actinic light was set to 100 ± 4 μmol photons m^−2^s^−1^ to generate a moderate level of photochemical quenching^45^. Effective quantum yield reflects the level of PSII activity under ambient (actinic) light conditions and is proportional to photosynthetic efficiency^48^. 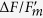 provides the most sensitive assessment of PSII herbicide impacts on photosynthesis at a given light intensity^47^. In order to calculate the maximum quantum yield of PSII (*F*_v_/*F*_m_), seagrass was dark-adapted for 30 min and *F*_*0*_ and *F*_*m*_ measured, as above, from *F*_*v*_/*F*_*m*_ = (*F*_*m*_ − *F*_*0*_)/*F*_*m*_. *F*_v_/*F*_m_ is a measure of the optimal photosynthetic efficiency and inhibition of *F*_v_/*F*_m_ can indicate photo-oxidative stress and damage to PSII from herbicide or thermal stress^48^.

### Temperature and diuron co-exposure experiments

Bioassays were conducted in incubators across a range of six temperatures (15–40 °C ± 0.5 °C) with 5 °C increments and diuron concentrations of 0, 0.3, 1, 3, 10, 30 μg l^−1^ at a light intensity of 100 ± 4 μmol photons m^−2^s^−1^ over a 14:10 h L:D diurnal cycle (LED light source). A preliminary range-finder experiment, indicated that 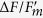 peaks at water temperatures near 30 °C for *H. ovalis* from Cockle Bay, Magnetic Island (confirmed during the study, see Results section). Hence, this temperature was designated as the “control” temperature. Prior to treatment exposures 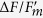 and *F*_*v*_/*F*_*m*_ were measured in leaves acclimated at 30 °C to provide initial PSII activity measurements for each leaf. Isolated leaves (n = 9 per treatment) were then transferred into well plates containing diuron and placed in an incubator at the appropriate treatment temperature. Replicates were randomly distributed across a set of nine well plates (per temperature treatment) and each temperature exposure was staggered by 50 min to allow time to measure 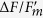 after fixed periods). 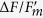 measurements were taken at 24 h post-exposure by transferring well plates onto a heating block under the I-PAM to maintain treatment temperature. An exposure period of 24 h was used as previous studies observed no decline in photosystem health of uncontaminated leaves over 48 h, and 24 h was long enough for diuron to induce maximum inhibition in isolated leaves^45^. Preliminary trials indicated that a steady state 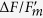 ~0.5 in control samples was reached in < 8 min, therefore, 8 minute actinic light acclimation periods were carried out for each plate prior to the recording of 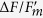 (see above). Samples were then dark adapted for 30 min and *F*_*v*_/*F*_*m*_ measurements were recorded. After 24 h exposure, all leaves were washed in fresh seawater and placed into new, uncontaminated 12-well plates with fresh seawater. Well plates were then placed in incubators set at 30 °C under the same illumination as described above. After a 24 h recovery period, 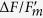 and *F*_*v*_/*F*_*m*_ measurements were recorded as described above.

### Data analysis

Temperature-only effects on 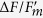 and *F*_*v*_/*F*_*m*_ were calculated from control leaf samples following 24 h exposure and a further 24 h recovery period. A three-parameter (maximum photosynthesis rate, thermal optima and maximum temperature for photosynthesis), model was fitted to the 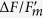 data in MATLAB and Statistics Toolbox Release R2016a (The MathWorks, Inc., Natick, Massachusetts, United States; https://www.mathworks.com/products/matlab/)^84^. Inhibition of quantum yields (% relative to solvent control) were calculated (Eq. 1) from treatment data. Inhibition of 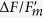 was calculated relative to the solvent control at 30 °C. Concentration-response curves were fitted using four-parameter logistic curves in GraphPad Prism v6 (San Diego, USA) using inhibition data at 24 h and after an additional 24 h recovery in fresh seawater at 30 °C. Diuron concentrations inhibiting 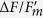 by 10% and 50% (IC_10_ and IC_50_) were determined from each curve by interpolation of the modelled curves (GraphPad Prism).


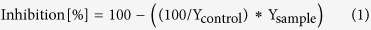


Where Y_control_ is 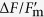 or *F*_*v*_/*F*_*m*_ of the solvent control and Y_sample_ is the 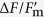 or *F*_*v*_/*F*_*m*_ of the treatment samples.

Predicted additive inhibition of 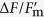 was calculated by applying the Response Addition (RA) equation (Eq. 2) to the data 55,69,85,86. This model is preferred when the modes of action of stressors are different (thermal inactivation of PSII and diuron binding to PSII) but the response being measured is common (inhibition of 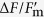 or *F*_*v*_/*F*_*m*_). The predicted inhibiton for additive responses from the RA model were plotted against the measured inhibition data for each combination of diuron and temperature relative to the 30 °C treatment in fresh seawater (solvent control).


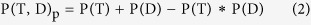


Where P(T,D)_p_ is the predicted additive effect of both variables tested; P(T) is the effect of temperature in the absence of diuron and P(D) is the effect of diuron at the control temperature, 30 °C. Both P(L) and P(T) are derived from raw data means.

If the experimental data falls on the 1:1 line (observed:predicted) then the combined effect of temperature is considered additive according to the RA model^55^,^69^,^86^. If the experimental data falls above the line, the effect is synergystic and if the data falls below the line the data is antagonistic or sub-additive. The combined effects of temperature and diuron on inhibition of 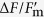 were also tested using a 2-way ANOVA on untransfomed % inhibition data (normality confirmed using the Shapiro-Wilk W test, NCSS V7, Kaysville, USA). The combined effects of temperature and diuron on inhibition of 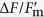 were also tested using a 2-way ANOVA on untransformed data for n = 9 replicate leaves per treatment (NCSS V7). The effect of temperature and diuron concentration on the inhibition of 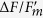 was modelled using a generalized additive model in R. The R code for this quasibinomial generalized additive model (GAM) best fit model can be found in Table S1. The 95% confidence intervals were based on the standard errors calculated by the predict.gam function (see “mgcv” package R documentation for details). The % recovery of individual leaves were calculated by comparing the *Fv*/*Fm* of individual leaves (n = 7–9 leaves replicate leaves per treatment) before the exposures and after 24 h recovery at 30 °C in clean seawater. The % recovery data was log_10_ transformed to achieve normal distribution according to the Shapiro-Wilk W test, and subjected to a 2-way ANOVA (NCSS V7).

## Additional Information

**How to cite this article**: Wilkinson, A. D. *et al*. Combined effects of temperature and the herbicide diuron on Photosystem II activity of the tropical seagrass *Halophila ovalis. Sci. Rep.* **7**, 45404; doi: 10.1038/srep45404 (2017).

**Publisher's note:** Springer Nature remains neutral with regard to jurisdictional claims in published maps and institutional affiliations.

## Supplementary Material

Supplementary Dataset 1

## Figures and Tables

**Table 1:** Diuron concentrations which inhibit 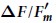 by 50% (IC_50_) at each temperature after 24 h exposures.

Temperature (°C)	IC_50_	95% CI	R^2^
15	NA	NA	0.82
20	2.7	2.0–3.5	0.86
25	2.6	2.2–3.1	0.94
30*	2.9	2.5–3.3	0.95
35	4.1	3.1–5.4	0.85
40	1.4	0–3.6	0.57

Inhibition of 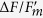 IC_50_ data (μg l^−1^ diuron) with 95% confidence intervals. *Control temperature optimum for 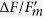. NA – the inhibition of 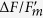 at 15 °C was over 50% in fresh seawater.

**Table 2:** Results from 2-way ANOVA on the effective quantum yields, 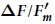 of *H. ovalis* exposed to varying concentrations of diuron at different temperatures after 24 h.

Factor	DF	SS	F	p
Temperature	5	1.257	63.79	<0.01
Diuron	5	6.099	309.64	<0.01
Temp x Diuron	25	0.605	6.14	<0.01
Error	286	1.127		

**Table 3:** Results from 2-way ANOVA on the recovery of maximum quantum yields, *Fv*/*F*_*m*_ of *H. ovalis* exposed to varying concentrations of diuron at different temperatures after 24 h and an additional 24 h in fresh seawater at 30 °C.

Factor	DF	SS	F	p
Temperature	5	0.0739	9.26	<0.01
Diuron	5	0.0631	7.9	<0.01
Temp × Diuron	25	0.0446	1.12	=0.32
Error	286	0.582		
